# Climate change, food security and health in Kiribati: a narrative review of the literature

**DOI:** 10.1080/16549716.2019.1603683

**Published:** 2019-05-07

**Authors:** John P. Cauchi, Ignacio Correa-Velez, Hilary Bambrick

**Affiliations:** a School of Public Health and Social Work, Queensland University of Technology, Kelvin Grove, QLD, Australia; b Institute of Health and Biomedical Innovation, Queensland University of Technology, Kelvin Grove, QLD, Australia

**Keywords:** Pacific, climate change, food security, health, Kiribati

## Abstract

**Background**: Climate change is recognised as having a ‘multiplier effect’ on food insecurity and adverse health experiences of communities in the Pacific region. Islands are especially at risk due to their limited land availability, population pressures and, in the case of atolls, their low-lying topography making them vulnerable to sea level rise.

**Aim**: This review examines the literature describing the relationship between climate change, food security and health in Kiribati.

**Method**: A narrative review was conducted, looking at both peer-reviewed and non-peer-reviewed literature available online from 1 January 2008 to 14 August 2018, the search date. Sources from three databases of peer-reviewed literature, Google and additional sources from reference lists were included in the review.

**Results**: Thirty-seven items were included in this review. These show climate change is having a noticeable impact on food security and health in Kiribati. Four themes were identified from the literature that provide different perspectives to the problem outlined.

**Conclusion**: Climate change is a pressing concern for the government of Kiribati and communities alike, and yet the problem is worsening, not improving. Further research is required to look at effective policies and cultural perspectives to address this problem.

## Background

Human health and well-being is dependent on the health and sustainability in the environmental context [1–3]. This is more pronounced in the context of a population living within the constraints of an island environment where the interaction between health and environment is more visible and experienced [4].

Recent research has identified growing public health concerns on the impact of climate change on health worldwide [2,5–7]. Pacific island countries (PICs) are among the most climate-vulnerable places on earth [8–10]. Faced with limited resources, population dynamics and in most cases a remoteness that offers its own challenges of limited connectivity to the rest of the world, their fragile socioecological context is further threatened by climate change. PIC climate vulnerability involves inherent risks, such as being low-lying, small landmass, and in the pathway of intense storms. Numerous publications and reports have outlined the situation of many small island developing states (SIDS) in the region which have been described as ‘canaries in the coal mine’ of climate change [9] many of which are poorly equipped to face the challenges ahead.

Rather than bringing new threats, climate change will largely affect population health by exacerbating existing problems, the so-called ‘multiplier effect’. In PICs, while some infectious diseases remain a significant burden, non-communicable diseases (NCDs) are a growing concern. The World Health Organisation (WHO) estimated in 2012 over 40% of 38 million NCD deaths globally were premature [11]. Tonga and Samoa, for example, have among the highest prevalence of obesity on earth [7,12]. Seventy-five percent of deaths in the Pacific region are due to NCDs [13]. This is often attributed to an increased dependence on imported food which is often of poor nutritional quality, inactive lifestyles and a shift towards a more Westernised diet [7,12,14].

### Why Kiribati?

Kiribati, the country with the lowest GDP per capita in the Pacific [15], can be described as a textbook case of inherent climate vulnerability with limited land area, overcrowding, low elevation of islands and water insecurity exacerbating public health problems [16]. In recent years, Kiribati has often made it to news headlines in relation to its vulnerability to climate change, with a narrative of islands being submerged this century due to sea level rise [17–20]. This has led the Government of Kiribati to purchase land in Fiji in order to ensure food security and a place for migration in the eventuality of sea level rise rendering their islands uninhabitable – a controversial decision not without its critics [21,22]. In this context of an existential threat, how can a country affected by so many challenges adapt to the ‘multiplier effects’ of climate change?

We will describe how food insecurity plays an important role in the rise of NCDs in the country. Both peer and non-peer reviewed literature on the topic will be used to specifically examine the following questions:

- What literature output of past 10 years exists linking climate change, food security and health in Kiribati?
- What reports or other forms of non-peer-reviewed literature in the past 10 years exist on the same topic?

The objective of this narrative review is to examine the existing literature on climate change and health impacts on what is often termed the ‘indirect’ or ‘second-tier’ level of climate change and health interaction [12], focusing on food security impacts on health in the Pacific atoll island-nation of Kiribati.

## Methods

### Retrieval

A systematic search was carried out on 14 August 2018 on three databases: PubMed, Web of Science and EBSCOhost. Google search was used for alternative sources on the same day. The Google search for non-peer-reviewed literature was opted for in light of the few items of peer-reviewed literature found in the databases above.

The search terms were related to climate change, food security, health and Kiribati. The full list of search terms for each database is provided in the Appendix. Items were selected if they were: 1) peer reviewed articles, or government and non-government reports exploring the topic with a considerable mention of Kiribati; 2) sources containing relevant information about at least two of the three key themes (i.e. climate change, food security, and health); 3) published in English; and 4) published between 1 January 2008 and 14 August 2018. See Appendix (Tables A1 and A2) for search terms used.

This review followed the steps outlined by PRISMA guidelines.

### Screening and eligibility

Following the execution of the search strategy, the identified items (titles and abstracts) were checked for duplication. Duplicates were removed. The unique items were then sorted according to type (e.g. articles, blogs, news articles, websites, etc.). The titles and abstracts of these unique items were then screened. Sources were excluded if 1) relevance to Kiribati was not sufficient; and 2) they were opinion pieces lacking empirical evidence.

**Figure 1.**
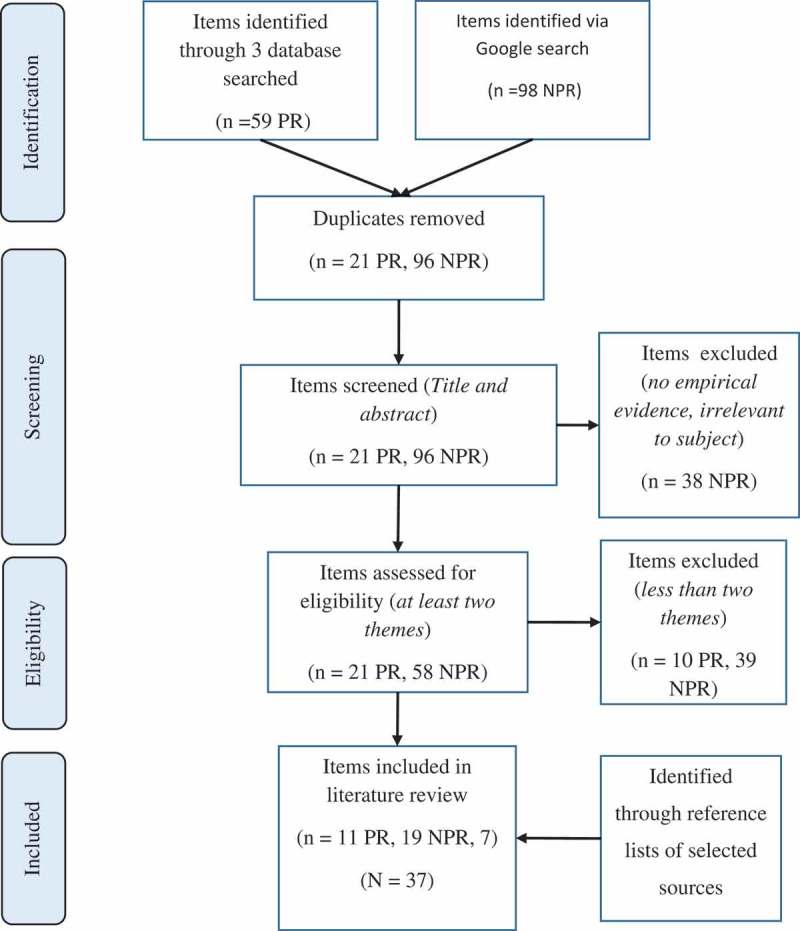
PRISMA selection process flow chart. * NPR = non peer reviewed (reports, blogs, videos, newspaper articles)* PR = Peer Reviewed (strictly articles in journals)

During this process, sources from reference lists and associated key items were assessed and additional new items were included in the final review (see Figure 1 below). If more recent versions of items (such as reports) were available, these were used for the purposes of this review.

### Narrative and thematic analysis

All selected items were then thoroughly reviewed to ascertain salient themes relating to the interaction of climate change, food security and health in Kiribati, which were then collated and described. Themes were determined depending on the extent to which they were discussed.

## Results

A total of 157 items were identified through the search strategy, which included peer reviewed articles from databases (n = 59) and other sources, mostly non-peer-reviewed literature (n = 98) via the Google search. Following the removal of duplicates, 117 unique items had their titles and abstracts screened for relevance, empirical evidence and mention of Kiribati. Exclusion here was defined by having a blog, for example, that focused on rural Kenya, or a blog post from a company that discussed food security purely from a commodity price perspective with barely any mention of climate change or health. Thirty-eight items were subsequently excluded.

The remaining items (n = 79) were assessed for depth of exploration of topic, where items that did not explore the interaction of at least two of the three themes of climate change, food security and health were excluded (for example, if an item only spoke about ‘climate change’ from a purely atmospheric science perspective, it was deemed not eligible). An additional 49 items were excluded at this stage. It is notable that no articles expressing doubt on links between food security problems and climate change were found during this process.

Any additional items identified during the eligibility process through references to reviewed items that met the eligibility criteria (n = 7) were then added to those to be included in the review. A thematic review was conducted on the final combination of items identified from databases (n = 11), Google search output (n = 19) and newly identified items (n = 7). Figure 1 shows a flow chart outlining the selection process for this systematic review. The items selected for review consisted of peer-reviewed articles and various other papers and reports (Table 1).

**Table 1.** Items included in review according to type and source.

Source	Peer Reviewed Literature	Non-peer reviewed literature (*including thesis, reports*)
Pubmed, EBSCO, Web of Science	11	0
Google Search	0	19
Sources from References	4	3

### Themes identified

Following the in-depth assessment of the selected items, four overarching themes were identified. The most common theme was the vulnerability of Kiribati in terms of urbanisation, environment and food security with 73% of items (n = 27) exploring this subject. The nutrition transition, lifestyle changes and impacts on public health were investigated by 68% of items (n = 25) while issues related to governance, communities and interventions were investigated in 46% (n = 17) of items. Lastly, 22% (n = 8) of items investigated the importance of traditional knowledge in ensuring food security in Kiribati. The items included in each theme are summarised in Table 2 below.

**Table 2.** Items sorted according to theme and type.

Theme/Focus	Peer-Reviewed Article	Report/Data Summary	Thesis
A – Vulnerability of Kiribati: Urbanisation, Environment and Food Security	11	16	0
B – The Nutrition Transition, Lifestyle changes and impacts on Public Health	12	13	0
C – Governance, Communities and Interventions	2	6	0
D – The Importance of Traditional Knowledge in Ensuring Food Security	6	11	1

#### Vulnerability of Kiribati: urbanisation, environment and food security (73%, n = 27)

Most of the items examining this theme were reports that stressed the inherent vulnerability of Kiribati on account of its geography, its population pressures and its problems in terms of environment, economics and isolation. Peer reviewed articles focused on the health impacts of climate change [16,23], and on food security and risks posed by climate change [24–30]. Migration and sociocultural issues impacted by climate change in Kiribati were also explored, further showcasing the complexity of the subject [31,32].

Non-peer-reviewed literature on the subject came in the form of reports (including the 2015 Kiribati census, with valuable statistics on areas of interest). Most reports found spoke of the problematic situation regarding food and nutritional security in Kiribati [33–39]. Lesley (2011) highlights the links of vulnerability in relation to poverty in Kiribati [5]. A 2016 United Nations University report highlighted the links between household vulnerability, migration and climate change in the country [40]. Interestingly, a report written by the Government of Kiribati in 2009 explores the security implications of climate change [41]. The 2007 National Adaptation programme of Kiribati, 2016 Kiribati Development Plan and the 2018 Voluntary National Review on Sustainable Development Goals of Kiribati fit in this category as they all recognise the vulnerability problems of Kiribati and the need to strengthen resilience [15,42,43]. Reports on the health impacts of climate change in Kiribati, linking to its vulnerability both locally and within the Pacific region context also explored this theme [44,45].

All items commonly highlighted the remoteness of the island nation, its inherent vulnerability due to its geography and problems related to water security and limits to its agricultural capacity. Urbanisation and population pressures on the environment of Kiribati were also mentioned as further factors that affect its inherent vulnerability. All items under this theme agree action is required in terms of increasing the resilience of Kiribati, ensuring food and water security and discussing the various impacts of urbanisation and population pressures, especially where the capital, South Tarawa, is concerned.

#### The nutrition transition, lifestyle changes and impacts on public health (68%, n = 25)

Most items in this theme explored the narrative of how urbanisation and lifestyle changes in Kiribati after the Second World War led to a nutrition transition that has negatively affected public health. Non-communicable diseases are now recognised as the leading cause of health problems in Kiribati.

Peer-reviewed articles included in this theme include two articles by McIver et al. with a predominantly health perspective, where Kiribati’s current dependence on imported food was linked to health outcomes [16,23]. Other articles noted that the current diet contributes strongly to micronutrient deficiencies, and importation of poor quality foods and lifestyles contributes to this problem [25,29,46,47]. A paper by Estimé et al. linked international trade as a structural driver of dietary risk factors in the Pacific [14]. Socio-cultural issues contributing to this problem were explored in depth by Weir et al. in 2017 [31]. Interestingly, two peer-reviewed articles in 2018 also explored the reactions of Kiribati crews on ships compared to their European counterparts where food and diet are concerned, which showed a clear difference in both nutritional knowledge and body perceptions of a desirable body shape [48,49]. Issues in food security adaptation projects as well as long-term implications of this problem were also explored by Cvitanovic et al. and Locke, respectively [32,50].

Non-peer-reviewed literature included many reports highlighting the issues related to globalisation and the poor-quality foods in Kiribati leading to considerable health problems. Statistics by WHO show alarming obesity prevalence of well over 45% and diabetes prevalence in over 23% of the population [51]. A WHO 2017 report on climate and health in Kiribati also outlines links between climate and food security, and mentions the impacts of Ciguatera poisoning in the country possibly linked to warmer seas [45]. Ciguatera is a foodborne illness brought about through the consumption of ciguatoxins (produced by dinoflagellate species such as *Gamberdiscus*) by reef fish. Its symptoms include diarrhoea and vomiting, paraesthesia, numbness, hot/cold sensitivity and general weakness. The toxins are not removed on cooking. Ciguatera seems to be of increasing concern in Kiribati, with three peer-reviewed articles exclusively focusing on the problem [27,28,30].

The 2015 Kiribati Census also highlights problems linked to the nutritional crisis in Kiribati. Valuable information on household assets, such as access to refrigeration and local food produce, paint a clearer picture of the degree of the problem [52]. Most reports, however, explore the problem from a regional perspective, with lifestyle changes noted in all countries in the region with the pattern of globalisation, poor economic performance, limited purchasing power and cultural preference for imported food over local food [5,33,37,38,44]. Biodiversity issues and food security problems as priority areas for future development in Kiribati were explored thoroughly by Government of Kiribati reports [15,35,36,42]. The Food and Agricultural Organisation (FAO) also published a food and nutrition security profile on Kiribati in 2014, outlining poor dietary diversity and an increased consumption of oils, sugar and carbohydrates that contributes to the high level of overweight prevalence (81.5% of adults) [53].

#### Governance, communities and interventions (46%, n = 17)

All articles and non-peer-reviewed literature under this theme noted considerable challenges in governance in Kiribati. Data is often incomplete, with ‘no data’ segments frequently visible on international reports by the WHO and FAO [51,53]. All articles on this theme pointed to salient problems in both the capacity of the government of Kiribati to manage the country, partly due to its vast geographic area, as well as cultural differences that lead to less-than-effective interventions. Cvitanovic et al. strongly explore this topic from a regional perspective, citing knowledge gaps in communities on how to adapt to climate change as well as a strong lack of trust between Pacific Island communities and adaptation scientists [50]. Betrand Noiret titles his article as a ‘Plea for ambitious action and inclusive development’ and sees an empowered civil society as key to effective interventions in food security [26]. Estimé makes the case for effective public health policy to be strongly linked to trade policy [14].

Lack of community involvement in effective climate adaptation points to strong challenges [14,31,54]. Prance examines the effectiveness of the Kiribati Adaptation Programme (KAP), the first of its kind, and the multitude of problems in its progress [54,55].

These problems are recognised by the Government of Kiribati in published reports [15,35,42,43,52]. Other international reports also point to enforcement difficulties in the region where problems such as overfishing or misuse of resources are concerned. Campbell and Hanich highlight this problem in fisheries development in Kiribati [34]. Others strongly emphasise the urgent need to address the food security problems in Kiribati while also recognising the limited capacity of the Government of Kiribati [33,36,38,44].

#### The importance of traditional knowledge in ensuring food security (22%, n = 8)

This theme includes considerable overlap with other themes. Items included here specifically highlight the importance of not only preserving but utilising traditional knowledge, and recognise it as key in future adaptation programmes. This is considered often from a resilience perspective, where using the knowledge carried down over centuries for enhancing food security is seen as a strong asset especially within communities in the Pacific including Kiribati [25,50].

The traditional diet is also showcased as a healthier option, with NCDs a result of moving away from such a diet and lifestyle towards Western diets which are noted to be key contributors to NCDs in the Pacific [44]. The Government of Kiribati recognises this, and it has included the need to value traditional knowledge both as a security priority as well as a key component of climate adaptation strategies [41,43].

## Discussion

This paper provides a comprehensive up-to-date narrative review of literature on climate change, food security and health in Kiribati. There is limited peer-reviewed literature that specifically examines this subject, with many articles linking at most two subject areas. This review therefore included non-peer-reviewed literature to enable a more comprehensive analysis of the situation in Kiribati.

### Summary of evidence

There is little research on the interaction of climate change and food security where Kiribati is concerned. While the link between food security and health is known and recognised, and the impact of climate change on this link clearly illustrated, there are few peer-reviewed articles that address the subject directly. There are, however, numerous reports from international organisations that describe this issue.

#### How does Kiribati fare within the context of the Pacific region?

Both peer-reviewed and non-peer-reviewed literature includes at least a mention of Kiribati within the context of the Pacific region, which is characterised by various remote, isolated countries with significant challenges in terms of infrastructure, population pressures and (with the notable exception of Papua New Guinea) limited land. McIver et al. compare different countries in the region in terms of the highest priority climate-sensitive health risks in individual Pacific island countries (see Table 3). Kiribati here is clearly not on its own regarding water, and food security issues, with a table showing that climate-sensitive health risks are found in all countries in the region. While it is exempt from extreme weather events such as cyclones on account of its geographical position, it is at risk of other events such as ‘king tides’ which are striking with increasing frequency [56]. Note also how ‘population pressures’ are significant enough in Kiribati to warrant their own entry, with health systems problems also listed as a mention (together with Fiji).

**Table 3.** Highest priority climate-sensitive health risks in individual Pacific Island countries (with each country’s highest priorities indicated by ‘x’).

Climate-Sensitive-Health-Risk	Country												
	Cook Islands	Fiji	Kiribati	Marshall Islands	Micronesia (Federated States)	Nauru	Niue	Palau	Samoa	Solomon Islands	Tonga	Tuvalu	Vanuatu
**Direct effects**													
Health impacts of extreme weather events	x	x		x	x	x	x	x	x	x	x	x	x
Heat-related illness	x					x	x			x			x
**Indirect effects**													
Water security & safety (including waterborne diseases)	x	x	x	x	x	x	x	x	x	x	x	x	x
Food security & safety (including malnutrition & foodborne diseases)	x	x	x	x	x	x	x		x	x	x	x	x
Vector-borne diseases	x	x	x	x	x	x	x	x	x	x	x	x	x
Zoonoses		x			x			x					
Respiratory illness	x			x	x	x	x	x		x		x	x
Disorders of the eyes, ears, skin and other body systems		x		x			x			x		x	x
**Diffuse effects**													
Disorders of mental/psychosocial health		x		x	x	x		x		x		x	x
Non-communicable diseases (NCDs)		x		x	x		x	x		x	x	x	x
Health systems problems		x	x										
Population pressures			x										

Source: modified from McIver et al., 2016 [9].

#### What do we know about the interaction of climate change, food security and health in Kiribati?

Kiribati is in a precarious food security situation. With a heavy and increasing dependence on imported food, combined with limited purchasing power, the people of Kiribati find themselves eating food of poor nutritional quality, leading to soaring levels of NCDs which are an urgent public health challenge [51]. While the agricultural output of Kiribati is clearly in decline especially in relation to its population growth [35–37], evidence shows the food insecurity experienced by most people in Kiribati, especially in urban South Tarawa (hosting half the population of Kiribati) is also due to lifestyle changes, cultural perception of foreign food and a lack of knowledge on nutrition. This is emphasised repeatedly, both in peer-reviewed articles and reports [16,24,36,40,45].

Although McIver et al. [9] did not identify NCDs as a high priority climate-sensitive health risk in Kiribati (see Table 3), data from the Global Burden of Disease study [57] shows high prevalence of diabetes, hypertension and heart disease. It could be argued that with less local food produced due to climate change, a heavier reliance on imported food, often of poor nutritional quality, is contributing to high levels of NCDs.

Kiribati has been described as a ‘canary in the coal mine’ for climate change effects [23]. Rising sea levels, salinisation of aquifers, coastal erosion, changing biodiversity, increasingly frequent ‘king tides’ and drought are repeatedly acknowledged as being on the increase, severely impacting both the socioeconomic and ecological environment of Kiribati [15,31,32,40,42]. Various interventions have been attempted over the years in order to increase the resilience of the people of Kiribati to these impacts, often with limited success [43,55]. Multiple reports also reveal how traditional knowledge is key to promoting nutritional diversity as well as biodiversity, in terms of both agriculture [35,36] and fishing [34].

These problems are exacerbated due to the limited capacity of the Government of Kiribati on account of both cultural differences and geographical and economic limitations, as well as a very ‘Western’ approach to solving challenges presented by climate change in international aid programmes and international institutions [54].

The evidence, however, also demonstrates that when traditional knowledge is valued and communities are involved as active participants, not as mere one-time consultants in order to ‘tick boxes’, projects can be remarkably successful as is witnessed by the Live & Learn Project carried out successfully in Temwaiku, South Tarawa [58]. In this project, the communities in Temwaiku collaborated on a joint food security project geared towards production of traditional foods of Kiribati and some non-indigenous foods. Every household was encouraged to have their own food garden as an adaptation to threats from climate change and food price volatility. This has provided some income, with part of the produce sold while significantly increasing the food security of the households involved.

However, while the problem has received some attention in peer-reviewed articles and extensively in non-peer-reviewed literature, little research exists outlining the community perceptions of climate change interactions on food security and health. This gap in knowledge is relevant, especially with the need for extensive adaptation to climate change being inevitable at this point.

#### Community interventions in Kiribati

Involving communities in food security interventions is a key component of any adaptation strategy. The Live & Learn Project in Temwaiku showcases such a successful intervention [58]. However, Prance clearly shows how a lack of dedicated community involvement led to less-than-successful outcomes in the KAP. Furthermore, a Western approach to problem-solving showcases a strong lack of willingness from institutional organisations to both understand the cultural differences in expectations and communication and the capacity of the Government of Kiribati in carrying out successful interventions [54,55]. This is a conclusion in agreement with Weir et. al, where, ‘Success is possible only with communities who recognise their need and are taking ownership and seeking help. Participation of the whole community in planning implementation is vital’ [31].

#### Future research

While the problems with food security and health in Kiribati and the probable impacts of climate change on this interaction are described, there is very little research on how households and communities make their food choices. Is it purely due to cultural reasons? Is it a matter of economic convenience? Is it perhaps that they would rather subsist on traditional food but have neither the time nor lifestyle to permit such a way of life in the 21st century? These questions remain mostly unanswered and are an opportunity for future research in this area which would help inform communities and policymakers on what could be an effective intervention while learning the lessons from KAP which faced numerous problems in this area.

#### Limitations of the study

This review has important limitations. First, the search process limited items to those in English-language databases. Secondly, while search strategies, exclusion criteria and analytical methods were discussed among co-authors in relation to the decision-making process, the items were selected by the first author. This could have introduced potential bias. Thirdly, reports on the subject are extensive, but it was limited to a 10-year period from 2008 to the present day for pragmatic reasons.

## Conclusion

Kiribati is highly vulnerable to the impacts of climate change in terms of food security and health. This is especially the case due to its inherent problems in terms of geography, population pressures and limited infrastructure. As climate change impacts increase, so will the problems in food security and health unless effective adaptation programmes are implemented. To date, interventions seem to have had little success.

This review concludes that rigorous evidence is scarce where Kiribati is concerned, especially regarding climate change, food security and health interactions. Numerous reports exist discussing the problem, but few outline successful interventions that can be replicated in Kiribati and elsewhere. Community perceptions of climate change and intervention projects in particular are needed so as to ensure effective and sustainable projects of this nature, especially in lieu of lessons learnt from the KAP. Given both the urgency of this subject and the vulnerability of Kiribati in particular, addressing this lack of information would be timely. This is especially the case as continued global greenhouse gas emissions mean some degree of climate adaptation is necessary in the coming decades. Additionally, greater cooperation between government agencies in Kiribati on climate risk is highly recommended, given the complexity in tackling problems in this area that often requires a multi-pronged approach. This could be achieved through the creation of an inter-agency initiative, for example, which could coordinate complex interventions requiring various sources of input.

## Paper context

Climate change is recognised as having a ‘multiplier effect’ on food insecurity and adverse health experiences of communities in the Pacific region. Islands are especially at risk due to their limited land availability, population pressures and, in the case of atolls, their low-lying topography making them vulnerable to sea level rise. Kiribati is considered a ‘textbook case’ for climate change studies due to its high vulnerability to climate impacts.

## Appendix Appendix

**Table A1.** Search terms for peer-reviewed articles.

Search #	Query	Items Found
**PUBMED**		
#1	*“Climate change“[All Fields] AND “Food security“[All Fields] AND “health“[All Fields] AND ”Kiribati”[All Fields] AND (”2008/01/01”[PDat]: ”2018/08/14”[PDat])*	0
#2	*“Climate change“[All Fields] AND “health“[All Fields] AND “Kiribati”[All Fields] AND (”2008/01/01”[PDat]: ”2018/08/14”[PDat])*	2
#3	*“Climate change“[All Fields] AND “food“[All Fields] AND “health“[All Fields] AND ”Kiribati”[All Fields] AND (”2008/01/01”[PDat]: ”2018/08/14”[PDat])*	1
#4	*“food“[All Fields] AND “health“[All Fields] AND “Kiribati”[All Fields] AND (”2008/01/01”[PDat]: ”2018/08/14”[PDat])*	7
**EBSCOHost (all databases)**		
#2	*(climate change or global warming) AND health AND Kiribati*. *Limiters – Published Date: 20080101–20181231; Hidden NetLibrary Holdings*	9
#3	*(climate change or global warming) AND food AND health AND Kiribati. Limiters – Published Date: 20080101–20181231; Hidden NetLibrary Holdings*	4
#4	*Food AND health AND Kiribati. Limiters – Published Date: 20080101–20181231; Hidden NetLibrary Holdings*	12
**Web of Science**		
#1	*TOPIC: (“Climate Change“ AND “Food Security“ AND ”health” AND ”Kiribati”)* *Indexes = SCI-EXPANDED, SSCI, A&HCI, CPCI-S, CPCI-SSH, BKCI-S, BKCI-SSH, ESCI, CCR-EXPANDED, IC Timespan = 2008–2018*	1
#2	*TOPIC: (“Climate Change“ AND “health” AND ”Kiribati”)* *Indexes = SCI-EXPANDED, SSCI, A&HCI, CPCI-S, CPCI-SSH, BKCI-S, BKCI-SSH, ESCI, CCR-EXPANDED, IC Timespan = 2008–2018*	6
#3	*TOPIC: (“Climate change“ AND “food“ AND ”health” AND ”Kiribati”)* *Indexes = SCI-EXPANDED, SSCI, A&HCI, CPCI-S, CPCI-SSH, BKCI-S, BKCI-SSH, ESCI, CCR-EXPANDED, IC Timespan = 2008–2018*	2
#4	*TOPIC: (“food“ AND “health” AND ”Kiribati”)* *Indexes = SCI-EXPANDED, SSCI, A&HCI, CPCI-S, CPCI-SSH, BKCI-S, BKCI-SSH, ESCI, CCR-EXPANDED, IC Timespan = 2008–2018*	9

**Table A2.** Search term for google search (searched on 14 August 2008).

Period of Time Search	Query (Search Term)	Items Found
1 January 2008–14 August 2018	*“Climate Change“ OR “global warming“ AND “Food“ OR “Food Security” OR ”Food Insecurity” AND ”health” AND ”Kiribati*”	98
